# Post-traumatic seizures and antiepileptic therapy as predictors of the functional outcome in patients with traumatic brain injury

**DOI:** 10.1038/s41598-021-84203-y

**Published:** 2021-02-25

**Authors:** Valeria Pingue, Chiara Mele, Antonio Nardone

**Affiliations:** 1 Neurorehabilitation and Spinal Unit, Institute of Pavia, Istituti Clinici Scientifici Maugeri IRCCS, Via Maugeri 4, 27100 Pavia, Italy; 2 Department of Clinical-Surgical, Diagnostic and Pediatric Sciences, University of Pavia, Pavia, Italy

**Keywords:** Drug discovery, Neuroscience, Health care, Neurology, Risk factors

## Abstract

Post-traumatic seizures (PTS) are a common and debilitating complication of traumatic brain injury (TBI) and could have a harmful impact on the progress of patient rehabilitation. To assess the effect of PTS and relative therapy on outcome in the initial phase after TBI, during the rehabilitation process when neuroplasticity is at its highest, we retrospectively examined the clinical data of 341 adult patients undergoing rehabilitation for at least 6 months post-TBI in our neurorehabilitation unit between 2008 and 2019. We correlated through logistic regression the occurrence of seizures and use of anti-seizure medication (ASM) with neurological and functional outcomes, respectively assessed with the Glasgow Coma Scale (GCS) and the Functional Independence Measure (FIM). PTS were documented in 19.4% of patients: early PTS (EPTS) in 7.0%; late PTS (LPTS) in 9.4%; both types in 3.0%. Patients who developed EPTS had an increased risk of developing LPTS (OR = 3.90, CI 95% 1.58–9.63, p = 0.003). Patients with LPTS had a significantly higher risk of worse neurological (p < 0.0001) and rehabilitation (p < 0.05) outcome. Overall, 38.7% of patients underwent therapy with ASM; prophylactic therapy was prescribed in 24.0% of patients, of whom 14.6% subsequently developed seizures. Mortality was associated with a lower FIM and GCS score on admission but not significantly with PTS. The use of ASM was associated with a worse rehabilitation outcome, independently of the onset of epilepsy during treatment. LPTS appear to exert a negative impact on rehabilitation outcome and their occurrence is not reduced by prophylactic therapy, whereas EPTS do not influence outcome. Our findings caution against the generic use of prophylactic therapy to prevent post-traumatic epilepsy in patients with TBI.

## Introduction

Patients surviving the early stages of traumatic brain injury (TBI) usually have a higher risk of developing disabilities and comorbidities later in life, and TBI has a severe impact on their life span. In this scenario, post-traumatic seizures (PTS) and post-traumatic epilepsy (PTE) are common and debilitating complications of TBI.

In relation to the time-frame of their occurrence, PTS are classified as “early” post-traumatic seizures (EPTS) if they occur within 7 days of the event, and “late” post-traumatic seizures (LPTS) if they occur > 7 days after the event^1,2^. This cut-off reflects differences in the causal mechanisms and subsequent seizure risk^3,4^. EPTS, also known as acute symptomatic seizures^5^, are linked to mechanisms of primary injury that temporarily lower the seizure threshold^4^. Instead, LPTS are characterized by persistent neurobiological changes attributed to secondary injury with biochemical cascades from epileptogenic mechanisms^6,7^ conditioning subsequent seizure risk^3,4^.

Considering the recent clinical redefinition of epilepsy from the International League Against Epilepsy (ILAE)^4^, the risk of recurrent seizures following a single, unprovoked seizure more than 7 days after TBI is high enough to consider LPTS as an epileptic condition. Therefore, the term LPTS is often used interchangeably with PTE^4,8^. The overall incidence of PTE in hospitalized patients is about 3–5%^9,10^, while it represents 10–20% of symptomatic epilepsy in the general population and 5% of all epilepsies^6^.

Seizures occurring during the acute care phase have a significant impact on the development of additional cerebral damage^11^. In particular, EPTS appear to increase morbidity and mortality in the early stages following TBI^12,13^ as well as the risk of developing PTE^14,15^.

Considering all these factors, early convulsive prophylaxis is commonly used after TBI in clinical practice, although with variable success^16^. For this reason, it has been a topic of research over the last few decades. While there is evidence of the effectiveness of anti-seizure medications (ASMs) in preventing EPTS, there is no proven benefit of ASM for LPTS and PTE^17–19^. In fact, the recent Brain Trauma Foundation Guidelines^20^ recommended the use of prophylactic therapy to decrease the incidence of EPTS within 7 days after severe TBI. Historically, phenytoin has been the ASM of choice as prophylactic therapy, but its complications have led to increasing use of levetiracetam as a substitute^21^*.* Even on this aspect, there is no clear evidence in the literature^22^. These inconsistencies could in part be due to the fact that previous epidemiological investigations on PTS were based on heterogeneous populations involving both adults and children^3,10,23^ and on large-scale multicentre databases where pre-existing epilepsy or previous neurologic injury were not excluded^15,24,25^.

To address these limitations, we selected only adult patients referred to our neurorehabilitation unit, excluding those with previous neurological conditions such as epilepsy. An advantage of the rehabilitative over the acute setting in assessing patients is the possibility of a more accurate evaluation thanks to the prolonged length of stay of patients. Therefore, in this study, we retrospectively analysed the clinical data of adult patients with TBI from the acute care phase and throughout the subsequent 6 months of inpatient rehabilitation. We focused our analysis within a 6-month period from injury since this period is crucial for the expression of neuroplasticity^26^. The primary aim of the study was to evaluate the impact of PTS and related antiepileptic drugs on neurological and functional outcomes after inpatient rehabilitation of post-TBI patients. The results of this analysis should give support to clinical decision-making regarding use of prophylactic anticonvulsant therapy in the initial phases after TBI.

## Methods

### Study design and population

In this observational retrospective study, we included all patients with TBI consecutively admitted to the Neurorehabilitation Unit of ICS Maugeri of Pavia, Italy between January 1, 2009 and December 31, 2018. Collection and analysis of clinical data were performed after approval by the ethics committee of ICS Maugeri (#2214 CE) and in accordance with the ethical standards laid down in the Declaration of Helsinki. Participants, or authorized representatives, signed a written informed consent before admission to neurorehabilitation unit.

The inclusion criteria were the following: (1) age ≥ 18 years; (2) diagnosis of TBI on presentation; (3) admission to a hospital emergency department within 24 h of injury; (4) admission within one month from the injury to the rehabilitation unit to continue clinical care and rehabilitation program; (5) up to 6 months of observation in the rehabilitation setting.

Individuals were excluded from the study if data regarding acute care were not available. We also excluded patients with pre-existing brain injury or other neurological diseases. Furthermore, patients with a history of epilepsy and concurrent use of ASM were not included.

### Variables, data sources and measurements

From patients’ hospital electronic records, we collected the following data: age at occurrence of injury, sex, medical history, injury characteristics, fracture site, presence of penetrating TBI, presence of subarachnoid haemorrhage, associated neurosurgical procedures (craniotomy, cranioplasty), neurologic and functional assessments, brain imaging, occurrence of seizures, presence and type of anticonvulsant therapy, death during hospitalization. Seizures were classified according to when they occurred, i.e. during acute care vs. rehabilitation phase. Finally, we collected data from the Glasgow Coma Scale (GCS) and the Functional Independence Measure (FIM) to evaluate, respectively, the neurological and functional outcomes. GCS is used not only to classify the severity of TBI and define its course, but it is also a validated predictor of clinical outcome after TBI^27–29^.

The overall GCS score ranges from 3 to 15; scores 3–8 indicate severe brain injury, 9–12 moderate brain injury, and 13–15 mild brain injury. FIM^30^ is an 18-item scale designed to measure the patient’s independence in activities of daily living. The severity of disability is evaluated with 13 motor (FIM-M) and 5 cognitive items (FIM-C). In our cohort, the GCS was administrated on arrival at the emergency department (GCS on Arrival; GCSoA). Both GCS and FIM were then administered at admission (T0) and discharge (T1) from the Neurorehabilitation Unit.

We also assessed the TBI characteristics, including type and location of the skull fracture, by radiological imaging. We used the adapted Marshall computed tomographic (CT) classification^31^ that categorizes injuries into six classes based on: degree of swelling as determined by basal cistern compression and midline shift, and presence and size of focal lesions (i.e. whether the lesion volume exceeds or not 25 cm^3^).

### Anti-seizure medication

Patients in treatment with ASMs were divided into two groups: those who were prescribed ASMs in acute care before the occurrence of seizure (prophylaxis group) vs. those who were prescribed ASMs after the onset of seizures in either the acute or rehabilitation setting (therapy for crisis group).

### PTS during acute care and inpatient neurorehabilitation

The presence of seizures during hospitalization was identified via medical records and classified based on time from injury into two classes as previously described^7,8^: 1–7 days after TBI (early) vs. > 7 days after TBI (late). Physicians examined any paroxysmal clinical event described by patients or eyewitnesses that occurred during hospitalization. Secondly, neurophysiological studies were performed for confirmation.

The documentation of clinical events and the administration of scales during rehabilitation were carried out by the same medical team. Data were collected by the first author and reviewed independently by the second author, with any discrepancies resolved by consensus.

### Statistical analysis

Values are expressed as median and interquartile range (IQR) or absolute number and percentage. Data were tested for normality of distribution with the Shapiro–Wilk test and log-transformed when needed in order to correct for skewness. Mann–Whitney and chi-square tests were used for comparisons between groups. Multinomial logistic regression analysis was performed to evaluate associations between presence of epilepsy or use of ASM and anthropometric data, clinical and radiological characteristics of TBI, rehabilitation outcome scores, and mortality. Multiple linear regression analysis was used to evaluate the predictive role of ASM therapy on rehabilitation outcome, adjusted for the presence of seizures. The multilinear models included FIM T1 or ΔFIM as dependent variables and ASM therapy and presence of seizures as independent variables. B coefficients, standard error (SE), β coefficients and significant values obtained from the models were reported.

A value of p < 0.05 was considered as statistically significant. All statistical analyses were performed using SPSS Statistics 21 (IBM Corporation, Somers, NY, USA).

### Ethical statement

The study was performed in accordance with the local institutional review board’s approvals.

### Consent for publication

All authors have approved the version to be published.

## Results

### Clinical characteristics of patients with TBI

**Figure 1:**
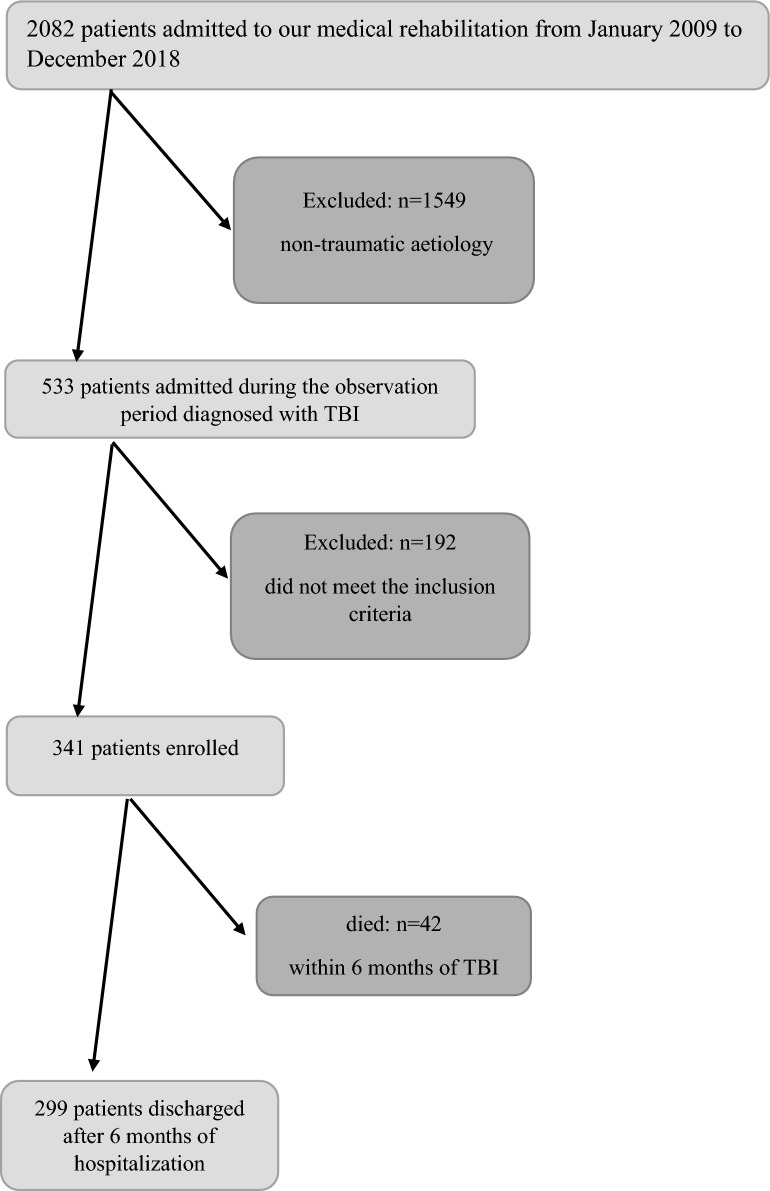
Consolidated standards of reporting trials (CONSORT)-like flow chart representing patient inclusion up to 6 months after traumatic brain injury (TBI).

A total of 2082 adult patients were admitted to our neurorehabilitation unit with a diagnosis of acquired brain injury from January 2009 to December 2018 (Fig. 1). Of these, 1549 patients (74.4%) were excluded because of a non-traumatic aetiology, and an additional 192 patients (9.2%) were excluded because they did not meet the other inclusion criteria. The remaining 341 adult patients with mild-to-severe TBI were enrolled in the study. Their demographic characteristics, post-traumatic clinical features and need for primary neurological and/or other surgery are reported in Table 1. Most patients (57.5%) were under 66 years of age at the time of TBI. The majority were males (78.0%), with a male:female ratio of 3.5:1.

**Table 1:** Demographic and clinical characteristics of patients with traumatic brain injury (TBI). Data for the whole group and subdivided according to TBI severity (mild, moderate and severe) based on the Glasgow Coma Scale on Arrival (GCSoA).

Variables	Whole population (n = 341)		TBI classification^&^ (GCS on Arrival—GCSoA) Data available for 270 patients						
	n	%	Mild (n = 32, 11.9%)		Moderate (n = 60, 22.2%)		Severe (n = 178, 65.9%)		p
			n	%	n	%	n	%	
**Age (years)**									
≤ 65	196	57.5	7	21.9	32	53.3	121	68.0	**< 0.0001**
> 65	145	42.5	25	78.1	28	46.7	57	32.0	
**Sex**									
M	266	78.0	22	68.7	52	86.7	140	78.7	0.12
F	75	22.0	10	31.3	8	13.3	38	21.3	
**Adapted Marshall** CT classification* ***Data available for: Whole sample = 336 patients***									
Diffuse injury I	16	4.8	2	6.2	5	8.4	4	2.3	0.10
Diffuse injury II	97	28.9	3	9.4	16	26.7	49	27.5	0.09
Diffuse injury III (swelling)	54	16.1	8	25.0	8	13.3	28	15.7	0.33
Diffuse injury IV (shift)	69	20.5	9	28.1	17	28.3	34	19.1	0.23
Evacuated lesion	100	29.8	10	31.3	14	23.3	63	35.4	0.22
Non evacuated lesion	0	0.0	0	0.0	0	0.0	0	0.0	–
**Subarachnoid haemorrhage**									
Yes	135	39.6	11	34.4	26	43.3	75	42.1	0.68
No	206	60.4	21	65.6	34	56.7	103	57.9	
**Lobar localization **^**§**^ ***Data available for:*** ***Whole sample = 273 patients*** ***Mild 30 patients*** ***Moderate 47 patients*** ***Severe 134 patients***									
Frontal	47	17.2	5	16.7	6	12.8	15	11.2	0.71
Parietal	16	5.9	0	0.0	0	0.0	3	2.3	0.42
Temporal	36	13.2	5	16.7	3	6.4	16	11.9	0.71
Occipital	6	2.2	0	0.0	2	4.2	2	1.5	0.37
Multiple	168	61.5	20	66.6	36	76.6	98	73.1	0.63
**Cranial fractures**									
Yes	170	49.9	14	43.8	29	48.3	100	56.2	0.31
No	171	50.1	18	56.2	31	51.7	78	43.8	
**Fracture site**									
Splanchnocranium	39	22.9	3	21.4	4	13.8	26	26.0	0.38
Skull base	12	7.1	3	21.4	3	10.3	5	5.0	0.08
Compound skull fracture	90	52.9	8	57.2	19	65.6	48	48.0	0.23
Depressed skull fracture	27	15.9	0	0.0	3	10.3	19	19.0	0.13
From blunt body	2	1.2	0	0.0	0	0.0	2	2.0	0.65
**Craniotomy**									
Yes	130	38.1	10	31.3	21	35.0	83	46.6	0.12
No	211	61.9	22	68.7	39	65.0	95	53.4	
**Cranioplasty**									
Yes	42	12.3	5	15.6	6	10.0	24	13.5	0.70
No	299	87.7	27	84.4	54	90.0	154	86.5	
**Post traumatic seizures (PTS)**									
Yes	66	19.4	6	18.8	9	15.0	39	21.9	0.50
No	275	80.6	26	81.2	51	85.0	139	78.1	
**Early post-traumatic seizure (EPTS)**									
Yes	24	7.0	1	3.1	4	6.7	13	7.3	0.53
No	317	93.0	31	96.9	56	93.3	165	92.7	
**Late post-traumatic seizure (LPTS)**									
Yes	32	9.4	2	6.3	5	8.3	20	11.2	0.67
No	309	90.6	30	93.7	55	91.7	158	88.8	
**Both seizures**									
Yes	10	2.9	3	9.4	0	0.0	6	3.4	0.06
No	331	97.1	29	90.6	60	100	172	96.6	
**Anti-seizure prophylactic therapy**									
Yes	82	24.0	7	21.9	16	26.7	47	26.4	0.85
No	259	76.0	25	78.1	44	73.3	131	73.6	
**Crisis therapy for seizures**									
Yes	50	14.7	5	15.6	8	13.3	27	15.2	0.93
No	291	85.3	27	84.4	52	86.7	151	84.8	
**Anti-seizure medication**									
None/not known	211	61.9	20	62.5	36	60.0	106	59.6	0.95
I generation drugs	32	9.4	4	12.5	2	3.3	20	11.2	0.17
II generation drugs	98	28.7	8	25.0	22	36.7	52	29.2	0.43
**Mortality within 6 months**									
Yes	42	12.3	5	15.6	8	13.3	26	14.6	0.95
No	299	87.7	27	84.4	52	86.7	152	85.4	

Significant difference are highlighted in bold.

Based on the GCSoA, TBI was mild in 11.9% cases, moderate in 22.2% and severe in 65.9%. As regards the localization of the trauma, most patients (61.5%) presented multiple site lesions, with frontal (17.2%) and temporal lobes (13.2%) being the most involved. As a consequence of the traumatic aetiology, approximately half of the patients (51.2%) presented skull fractures, mostly compound skull fractures. In 39.6% of patients, the presence of subarachnoid haemorrhage was detected. Regarding neurosurgical interventions, 38.1% of patients underwent craniotomy and 12.3% cranioplasty. A significant difference between the three classes of TBI severity was found only for age at diagnosis. In fact, patients with moderate and severe TBI were significantly (p < 0.0001) younger compared to those with mild TBI.

### Clinical and therapeutic aspects of TBI patients who experienced seizures

During the observation period from acute care to inpatient rehabilitation, 66 patients (19.4%) had reported or documented seizure activity. EPTS were documented in 24 cases (7.0%), LPTS in 32 cases (9.4%) while 10 patients (3.0%) first presented EPTS and then LPTS, two of them being on antiepileptic prophylactic therapy. The clinical and therapeutic characteristics of patients with seizures are reported in Table 1.

Overall, 132 patients (38.7%) were prescribed ASM. It was prescribed as prophylactic therapy in 82 patients (24.0%), 10 (14.6%) of which subsequently developed seizures (1 EPTS, 9 LPTS and 2 both types). It was prescribed as treatment for crisis in 50 patients (12.2%). Most patients (74.2%) received levetiracetam (II generation ASM). There were no severe drug-related toxic effects during hospitalization and in both groups ASMs were continued for the rest of patients’ stay in the rehabilitation unit. During the 6-month observation period, 42 patients (12.3%) died, but only 6 of them (14.3%) had experienced epilepsy.

Compared to patients without seizures, those who developed seizures were more frequently found to have an evacuated mass lesion according to the adapted Marshall classification (p < 0.01), and a higher prevalence of compound skull fracture (p < 0.05) and they more frequently underwent craniotomy (p < 0.01) and/or cranioplasty (p < 0.05) (Table 2).

**Table 2:** Comparison of traumatic brain injury (TBI) patients without vs. with post-traumatic seizures (PTS), and between patients with early PTS (EPTS) vs. late PTS (LPTS) vs. both EPTS + LPTS.

Variables	Without PTS (n = 275)		With PTS (n = 66)		EPTS (n = 24)		LPTS (n = 32)		Both (EPTS + LPTS) (n = 10)	
	n	%	n	%	n	%	n	%	n	%
**Age (years)**										
≤ 65	158	57.5	38	57.6	12	50.0	21	65.6	5	50.0
> 65	117	42.5	28	42.4	12	50.0	11	34.4	5	50.0
**Sex**										
M	216	78.5	50	75.8	18	75.0	26	81.3	6	60.0
F	59	21.5	16	24.2	6	25.0	6	18.7	4	40.0
**TBI classification**^**&**^ **(GCS on Arrival)** ***Data available for:*** ***Without PTS = 216 patients*** ***With PTS = 54 patients*** ***EPTS = 18 patients*** ***LPTS = 27 patients*** ***EPTS + LPTS = 9 patients***										
Mild	26	12.0	6	11.1	1	5.6	2	7.4	3	33.3
Moderate	51	23.6	9	16.7	4	22.2	5	18.5	0	0.0
Severe	139	64.4	39	72.2	13	72.2	20	74.1	6	66.7
**Adapted Marshall** classification* ***Data available for:*** ***Without PTS = 272 patients*** ***PTS = 64 patients*** ***LPTS = 30 patients***										
Diffuse injury I	16	5.9	0	0.0	0	0.0	0	0.0	0	0.0
Diffuse injury II	81	29.8	16	25.0	4	16.7	9	30.0	3	30.0
Diffuse injury III (swelling)	45	16.5	9	14.1	**7**	**29.1**	**2**	**6.7**^**e**^	0	0.0
Diffuse injury IV (shift)	57	21.0	12	18.7	4	16.7	6	20.0	2	20.0
Evacuated mass lesion	**73**	**26.8**	**27**	**48.2**^**b**^	9	37.5	13	43.3	5	50.0
Non evacuated mass lesion	0	0.0	0	0.0	0	0.0	0	0.0	0	0.0
**Subarachnoid haemorrhage**										
Yes	107	38.9	28	42.4	10	41.7	13	40.6	5	50.0
No	168	61.1	38	57.6	14	58.3	19	59.4	5	50.0
**Lobar localization**^**§**^ ***Data available for:*** ***No PTS = 198 patients*** ***PTS = 49 patients*** ***EPTS = 20 patients*** ***LPTS = 22 patients*** ***EPTS + LPTS = 7 patients***										
Frontal	28	14.1	9	18.3	4	20.0	2	9.1	3	42.8
Parietal	**6**	**3.0**	2	4.1	0	0.0	0	0.0	**2**	**28.6**^**a**^
Temporal	25	12.6	4	8.2	2	10.0	2	9.1	0	0.0
Occipital	3	1.5	2	4.1	1	5.0	1	4.5	0	0.0
Multiple	**136**	**68.8**	32	65.3	13	65.0	17	77.3	**2**	**28.6**^**a**^
Cranial fractures										
Yes	138	50.2	32	48.5	10	41.7	17	53.1	5	50.0
No	137	49.8	34	51.5	14	58.3	15	46.9	5	50.0
**Fracture site**										
Splanchnocranium	33	23.9	6	18.7	0	0.0	5	29.4	1	20.0
Skull base	11	8.0	1	3.2	1	10.0	0	0.0	0	0.0
Compound skull fracture	**68**	**49.3**	**22**	**68.7**^**a**^	**9**	**90.0**^**b**^	**9**	**52.9**^**e**^	4	80.0
Depressed skull fracture	25	18.1	2	6.2	0	0.0	2	11.8	0	0.0
From blunt body	1	0.7	1	3.2	0	0.0	1	5.9	0	0.0
**Craniotomy**										
Yes	**95**	**34.5**	**35**	**53.0**^**b**^	12	50.0	**17**	**53.1**^**a**^	6	60.0
No	180	65.5	31	47.0	12	50.0	15	46.9	4	40.0
**Cranioplasty**										
Yes	**29**	**10.5**	**13**	**19.7**^**a**^	2	8.3	**9**	**28.1**^**b**^	2	20.0
No	246	89.5	53	80.3	22	91.7	23	71.9	8	80.0
**Anti-seizure prophylactic therapy**										
Yes	**72**	**26.2**	10	15.2	**1**	**4.2**^**a**^	**9**	**28.1**^**e**^	0	0.0
No	203	73.8	56	84.8	23	95.8	23	71.9	10	100
**Therapy for seizures**										
Yes	**0**	**0.0**	**50**	**75.8**^**d**^	**18**	**75.0**^**d**^	**22**	**68.8**^**d**^	**10**	**100**^**d**^
No	275	100	16	24.2	6	25.0	10	31.2	0	0.0
**Anti-seizure medication**										
None/not known	**203**	**73.8**	**8**	**12.1**^**d**^	**7**	**29.2**^**d**^	**1**	**3.1**^**d,e**^	0	0.0
I generation drugs	**15**	**5.5**	**17**	**25.8**^**d**^	3	12.5	**12**	**37.5**^**d**^	2	20.0
II generation drugs	**57**	**20.7**	**41**	**62.1**^**d**^	**14**	**58.3**^**c**^	**19**	**59.4**^**d**^	**8**	**80.0**^**c**^
**Mortality within 6 months**										
Yes	36	13.1	6	9.1	3	12.5	3	9.3	0	0.0
No	239	86.9	60	90.9	21	87.5	29	906	10	100

Data are expressed as absolute number and percentage. Comparisons between groups were performed with χ^2^ test.

Significant differences between patients without vs. with epilepsy or between EPTS and LPTS or both (EPTS + LPTS) are expressed as ^a^p < 0.05, ^b^p < 0.01, ^c^p < 0.001, ^d^p < 0.0001.

Significant differences between patients with EPTS vs. LPTS are expressed as ^e^p < 0.05, ^f^p < 0.01.

Significant difference are highlighted in bold.

We analysed patients with epilepsy according to the type of seizure (EPTS or LPTS or both). Compared to LPTS, patients with EPTS had a significantly higher frequency of diffuse injury grade III according to the adapted Marshall classification and of compound skull fracture (p < 0.05). Moreover, EPTS patients had a significantly higher proportion of compound than depressed skull fractures compared to patients without epilepsy (p < 0.01). On the other hand, patients with LPTS had more frequently undergone craniotomy (p < 0.05) and cranioplasty (p < 0.01), and had a lower CGS (p < 0.05) and FIM (p < 0.05) on discharge.

Finally, comparing patients according to TBI severity, despite the small sample size of patients with mild and moderate TBI, the same differences as above were observed in each group of TBI severity (not shown).

### Association between clinical aspects of TBI and risk of seizures

A multinomial logistic regression analysis was conducted to evaluate the association between the clinical aspects of TBI and the risk of seizure onset. All association analyses were weighted for age, gender and severity of TBI. Patients who underwent craniotomy or cranioplasty had a higher risk of seizures than those who did not undergo these interventions (OR = 2.12, CI 95% 1.24–3.66, p = 0.007; and OR = 2.07, CI 95% 1.01–4.25, p = 0.047, respectively), in particular a higher risk of LPTS onset (OR = 2.16, CI 95% 2.13–4.15, p = 0.02; and OR = 3.06, CI 95% 1.40–6.68, p = 0.005, respectively).

We did not find any association between the clinical aspects of TBI and EPTS onset (data not shown). Of note, patients who developed EPTS had an increased risk of developing LPTS (OR = 3.90, CI 95% 1.58–9.63, p = 0.003). Patients treated with ASM had a significantly lower risk of EPTS onset (OR = 0.10, CI 95% 0.01–0.76, p = 0.03). But they did not show a significantly lower risk of developing LPTS (OR = 0.69, CI 95% 0.28–1.65, p = 0.40) or any PTS (OR = 0.50, CI 95% 0.24–1.15, p = 0.12).

### Neurological and rehabilitation outcome

At multinomial logistic regression analysis, the presence of seizures was associated with a worse score on GCS (p < 0.05) and FIM (p < 0.01) at the end of inpatient rehabilitation (Table 3).

**Table 3:** Association between presence of post-traumatic seizures (PTS) and neurological or rehabilitation outcome, respectively measured with the Glasgow Coma Scale (GCS) and the Functional Independence Measure (FIM) at 6 months from traumatic brain injury**.** Odds ratios (OR) are given for the overall group of patients with PTS and for patients subdivided according to early (EPTS) vs. late PTS (LPTS).

Covariates	PTS (no = 0, yes = 1)			Early-PTS (no = 0; yes = 1)			Late-PTS (no = 0; yes = 1)		
	OR	CI 95%	p	OR	CI 95%	p	OR	CI 95%	p
GCS T0	0.88	0.62–1.26	0.50	1.16	0.72–1.86	0.55	**0.61**	**0.39–0.93**	**0.02**
GCS T1	**0.69**	**0.49–0.97**	**0.03**	1.14	0.70–1.85	0.59	**0.49**	**0.33–0.73**	** < 0.0001**
ΔGCS	0.82	0.60–1.12	0.21	0.96	0.65–1.42	0.86	0.80	0.55–1.18	0.26
FIM T0	0.99	0.98–1.00	0.22	0.99	0.97–1.01	0.26	0.99	0.98–1.01	0.23
FIM T1	**0.99**	**0.98–0.99**	**0.007**	0.99	0.97–1.00	0.06	**0.99**	**0.98–0.99**	**0.019**
ΔFIM	**0.98**	**0.97–0.99**	**0.003**	0.99	0.97–1.00	0.06	**0.98**	**0.97–0.99**	**0.016**

*T0* on admission to neurorehabilitation, *T1* at discharge.

Significant difference are highlighted in bold.

Patients with LPTS had a significantly higher risk of worse neurological (p < 0.0001) and functional (p < 0.05) outcomes than those with EPTS, in whom this risk did not reach statistical significance.

Further analyses were conducted to compare neurological/rehabilitation outcome parameters between patients according to the use or not of ASMs. As shown in Table 4, patients treated with ASMs had a worse neurological outcome than those not treated.

**Table 4:** Neurological/rehabilitation outcome measured with the Glasgow Coma Scale (GCS) and the Functional Independence Measure (FIM) in patients according to the use or not of ASMs.

Variables	Patients treated with ASMs	Patients not treated	p-value
	Median (IQR)	Median (IQR)	
GCS T0	10 (8–12)	11 (9–13)	0.07
GCS T1	13 (11–15)	14 (12–15)	0.12
ΔGCS	2 (0–4)	1 (0–3)	0.10
FIM T0	**18 (18–26)**	**26 (18–67)**	**0.001**
FIM T1	**23 (18–79)**	**90 (20–119)**	**< 0.0001**
ΔFIM	**18 (0–36)**	**31 (0–60)**	**< 0.0001**

*T0* on admission to neurorehabilitation, *T1* at discharge.

Data are expressed as median and interquartile range. Comparison between groups was performed using Mann–Whitney test. Significant differences are shown in bold.

To further explore the relationship between the use of ASM and outcome, multinomial logistic regression analyses were conducted (Table 5).

**Table 5:** Odds ratios (ORs) for the association between the use of anti-epileptic drugs and neurological/rehabilitation outcome measured with the Glasgow Coma Scale (GCS) and the Functional Independence Measure (FIM).

Covariates	Prophylactic therapy (no = 0; yes = 1)						Therapy for seizures (no = 0; yes = 1)			Type of medication (I generation = 0; II generation = 1)		
	Not PTS			PTS								
	OR	CI 95%	p	OR	CI 95%	p	OR	CI 95%	p	OR	CI 95%	p
GCS T0	**0.51**	**0.35–0.73**	**0.0001**	1.05	0.43–2.56	0.92	0.79	0.54–1.17	0.25	1.01	0.59–1.72	0.97
GCS T1	**0.64**	**0.45–0.92**	**0.02**	1.64	0.63–4.25	0.31	0.78	0.45–1.00	0.07	0.83	0.52–1.34	0.45
ΔGCS	1.13	0.85–1.50	0.40	1.35	0.69–2.64	0.38	0.84	0.60–1.20	0.34	0.86	0.54–1.34	0.51
FIM T0	**0.98**	**0.97–0.99**	**0.004**	1.01	0.99–1.04	0.21	0.99	0.97–1.00	0.06	0.99	0.97–1.00	0.08
FIM T1	**0.99**	**0.98–0.99**	**0.001**	1.01	0.99–1.03	0.34	**0.99**	**0.98–0.99**	**0.003**	0.99	0.98–1.01	0.41
ΔFIM	**0.99**	**0.98–1.00**	**0.04**	0.98	0.95–1.02	0.31	**0.99**	**0.97–0.99**	**0.02**	1.00	0.99–1.02	0.83

*T0* on admission to neurorehabilitation, *T1* at discharge.

Significant difference are highlighted in bold.

Concerning the use of prophylactic therapy, we grouped patients according to onset or absence of seizures during anticonvulsant treatment, with the aim to evaluate whether the association with worse rehabilitation outcome was linked only to the presence of seizures or also to the effect of ASM. Our results showed that the use of ASM, either as a prophylactic or for crisis therapy, regardless of the onset of epilepsy during treatment, was associated with a significantly worse FIM (Table 5). Multiple linear regression analysis (adjusting for the presence of seizures) confirmed that both ASM use and PTS independently predicted rehabilitation outcomes (FIMT1 and ΔFIM) and that the association between FIM and ASM was independent of the presence of PTS (Tables 6 and 7).

**Table 6:** Multiple linear regression analysis showing independent predictors for FIM at discharge (T1).

Model dependent variable: FIM T1	Unstandardized coefficients		Standardized coefficients	*t*	p-value
	B	SE	Beta		
Constant	63.79	4.32	–	14.76	< 0.0001
Prophylactic therapy (no = 0; yes = 1)	− 19.95	7.56	− 0.19	− 2.64	0.009
PTS (no = 0; yes = 1)	− 21.52	8.52	− 0.18	− 2.53	0.012

*FIM* functional independence measure, *PTS* post-traumatic seizures, *T1* at discharge.

**Table 7:** Multiple linear regression analysis showing independent predictors for ΔFIM.

Model dependent variable: ΔFIM	Unstandardized coefficients		Standardized coefficients	*t*	p-value
	B	SE	Beta		
Constant	29.44	3.27	–	9.00	< 0.0001
Prophylactic therapy (no = 0; yes = 1)	− 11.81	5.70	− 0.15	− 2.07	0.04
PTS (no = 0; yes = 1)	− 17.50	6.36	− 0.20	− 2.75	0.007

*FIM* functional independence measure, *PTS* post-traumatic seizures, *T1* at discharge.

We did not find any association between the type of medication used (I or II generation) and neurological or rehabilitative outcome. The same associations between the use of ASM and outcome were found when patients were subdivided according to TBI severity based on the GCSoA (data not shown).

### Mortality

Mortality at 6 months from TBI was documented in 42 patients (12.3%) (Table 1). There was no significant difference in prevalence of mortality between the three classes of TBI severity. Lower FIM and GCS scores on admission were associated with higher risk of mortality at 6 months from TBI (OR = 0.94, CI 95% 0.90–0.98, p < 0.01; and OR = 0.39, CI 95% 0.25–0.61, p < 0.0001, respectively). Moreover, mortality was higher in patients > 65 years of age (OR = 8.6, CI 95% 3.71–19.92, p < 0.0001) and in patients who had an evacuated mass lesion on the adapted Marshall Classification (OR = 5.1, CI 95% 1.69–15.56, p < 0.01). We did not find a significant association between mortality and the presence of epilepsy or the use of ASM (data not shown).

## Discussion

### Incidence and risk factors of PTS

In this study, we evaluated the impact of PTS and relative antiepileptic therapy on neurological and functional outcomes in a large sample of adult patients undergoing rehabilitation after mild to severe TBI and followed for up to 6 months after injury. The incidence of EPTS and LPTS we observed was higher than that found in an earlier study of 1998^10^, but in line with another more recent population-based study^15^ which had a similar patient profile in terms of TBI severity. The difference in frequency of overall PTS with the earlier study could be explained by the fact that nowadays more patients with moderate-severe TBI survive after severe brain injury. The increasing use of electroencephalography monitoring during acute care also enables clinicians to detect more precisely any type of seizure, thus increasing the diagnostic sensitivity.

Risk factors for PTS have been widely described in the literature^15,32,33^. In our cohort, the overall risk of PTS was strongly associated with injury characteristics (evacuated mass lesion, compound skull fracture) and neurosurgical procedures (craniotomy and cranioplasty). In particular, patients with a grade III Marshall Classification and compound skull fractures had a higher rate of EPTS, while craniotomy and cranioplasty procedures were significantly associated with LPTS.

Analyzing the associations between clinical aspects of TBI and PTS risk, we found a strong correlation between LPTS and neurosurgery procedures, consistent with the existing literature^3,14,15,24,34,35^. In line with our findings, a recent study conducted in a paediatric population demonstrated an effect of neurosurgery procedures on seizure risk within the first 6 months after trauma^36^. Instead, no association was found between the clinical variables and EPTS, although patients who had EPTS had an increased risk of developing LPTS, presumably due to the primary mechanical injury characteristics. We found no age- or sex-related difference regarding the risk of developing PTS at 6 months. Similarly to Rittel et al.^15^, no correlation between PTS and TBI severity was detected. It is important to underline that our study did not include individuals who were not hospitalized after trauma, possibly limiting the sensitivity of detecting PTS. Finally, in our study, risk of mortality was significantly associated to lower FIM and GCS scores at admission, without any correlation with PTS at 6 months after the event.

### Neurological and functional outcomes related to seizures

In terms of recovery after inpatient rehabilitation, only LPTS and use of ASM were significantly related to worse neurological and rehabilitative outcomes, whereas EPTS did not negatively affect the outcome. These findings are consistent with a previous study that demonstrated no difference in neurological recovery at 6 months between patients with or without EPTS^23^. In our cohort, the occurrence of LPTS was not related to the severity of TBI measured with the GCS on arrival in the emergency department. However, LPTS negatively influenced neurological and functional outcomes at 6 months from trauma, when neuroplasticity is at its highest^26^. To date a large number of studies have demonstrated that after TBI the primary injury is followed by a cascade of metabolic, biochemical and inflammatory changes^36–38^. These events trigger secondary brain injury resulting in delayed neuronal loss and abnormal neuronal excitability that influence the long-term TBI complications, such as epilepsy^36,39,40^. This pathological process can also impair the regenerative process after brain injury^11^, influencing neurological and functional outcome.

### Seizures and post-traumatic epilepsy in relation to ASM use

The 2016 guidelines for the management of severe TBI from the Brain Trauma Foundation and the American Association of Neurological Surgeons^20^ suggest that patients with severe TBI may be treated with ASM soon after trauma to prevent EPTS in that the overall benefits outweigh the risks associated with treatment. These recommendations are heavily based on research by Temkin et al. (1990)^14^ who demonstrated a significant reduction in the incidence of EPTS with phenytoin compared to placebo^14^. In this context, an interesting finding of our study is that, although we confirmed that the prophylactic use of any ASM in the 6 months following TBI actually seems to have a protective effect on EPTS, this therapy does not reduce the risk of LPTS. This finding supports the hypothesis that anticonvulsant prophylaxis does not prevent epileptogenic mechanisms coming into play after TBI^41,42^. Indeed, these mechanisms that may contribute to PTE are still poorly understood, making treatment with ASM of little effect in preventing PTE^11,43^.

Recent studies have demonstrated no evidence that early treatment with ASMs reduces the risk of LPTS or mortality, whereas it seems to adversely affect functional outcome in the long term^14,19,33,41,44–46^. Levetiracetam, used for early post-TBI seizure prophylaxis, seems to be associated with a shorter length of hospital stay than phenytoin^21^.

In our cohort, ASMs seem to prevent EPTS but not LPTS, supporting the hypothesis that EPTS and LPTS may have different causal mechanisms^39,40^. Indeed, EPTS appear to be directly related to the primary mechanical injury, whereas LPTS are a consequence of the secondary process that begins a few minutes after head injury and can persist for months or years^39^. Moreover, our data showed that the use of antiepileptic drugs appears to be associated with a worsening of functional outcomes, independently of whether therapy is I or II generation and of the occurrence or not of seizures. No toxicity or serious events related to I and II generation ASMs were reported during the observation period. Indeed, in line with recent reviews, our study confirms the same efficacy and safety for both medications with regards to early and late seizure prophylaxis following TBI^22^.

In summary, our findings raise a further warning against the generic use of prophylaxis with ASM for PTE in the initial phase after TBI. ASM should indeed be limited to those clinical conditions at high risk of LPTS such as patients who have to undergo neurosurgical procedures or, as recommended by the Brain Trauma Foundation guidelines, they should be limited to 7 days after severe head injury.

### Study limitations

This study has several limitations mainly due to its retrospective nature. The dataset we used in this study is claim-based and susceptible to miscoding and missing information. In particular, a major limitation is the fact that different protocols were used for the administration of prophylactic therapy in acute care. In this setting, the ASMs were prescribed at physicians’ discretion, probably in relation to the severity of the case. Indeed, our study could not differentiate the use of ASMs in relation to TBI severity or to a clinical indication. This is an inherent confounding factor that requires prospective studies to better understand the effects of ASMs on rehabilitation outcomes.

Another limitation is the high rate of patients with severe TBI in the cohort*.* However, comparison analysis was conducted in each group of TBI severity and, despite the low sample size of patients with mild or moderate trauma, the same differences as above were observed in each group. Finally, the observation period was relatively short compared to other studies, but our goal was to verify the implications of seizures and epilepsy during the inpatient rehabilitation process and not beyond.

## Conclusions

The occurrence of LPTS during rehabilitation negatively influences patient outcomes, and the use of ASM does not seem effective in preventing them. In contrast, prophylactic ASM is more effective in preventing EPTS and appears to have no impact on neurological and functional outcomes and on the risk of late seizures. This study underlines the need to re-examine the use of prophylactic ASM for the prevention of PTE. Based on this retrospective study, prescription of prophylactic ASM after TBI should be limited to those conditions that show evidence of high risk of LPTS or otherwise used for a few days after severe head injury.
